# Study on the Effects of Quantum Well Location on Optical Characteristics of AlGaN/GaN Light-Emitting HEMT

**DOI:** 10.3390/mi14020423

**Published:** 2023-02-10

**Authors:** Yao-Luen Shen, Chih-Yao Chang, Po-Liang Chen, Cheng-Chan Tai, Tian-Li Wu, Yuh-Renn Wu, Chih-Fang Huang

**Affiliations:** 1 Institute of Electronics Engineering, National Tsing Hua University, Hsinchu 30010, Taiwan; 2 International College of Semiconductor Technology, National Yang Ming Chiao Tung University, Hsinchu 30010, Taiwan; 3 Department of Electrical Engineering, National Taiwan University, Taipei 10617, Taiwan

**Keywords:** GaN, light-emitting, HEMT, monolithic integration, single quantum well, simulation

## Abstract

In this study, AlGaN/GaN light-emitting HEMTs (LE-HEMT) with a single quantum well inserted in different locations in the epitaxy layers are fabricated and analyzed. For both structures, light-emitting originated from electrons in the 2DEG and holes from the p-GaN for radiative recombination is located in the quantum well. To investigate the importance of the location of single quantum well, optical characteristics are compared by simulation and experimental results. The experimental results show that the main light-emitting wavelength is shifted from 365 nm in the UV range to 525 nm in the visible range when the radiative recombination is confined in the quantum well and dominates among other mechanisms. Epi B, which has a quantum well above the AlGaN barrier layer in contrast to Epi A which has a quantum well underneath the barrier, shows better intensity and uniformity in light-emitting. According to the simulation results showing the radiative distribution and electron concentrations for both structures, the lower quantum efficiency is due to the diverse current paths in Epi A. On the other hand, Epi B shows better quantum confinement and therefore better luminescence in the same bias condition, which is consistent with experimental observations. These findings are critical for advancing the performance of LE-HEMTs.

## 1. Introduction

Gallium nitride-based devices have been widely used mainly in two types of applications. One is high electron mobility transistors (HEMTs) for electronical devices and the other is light-emitting diodes (LEDs) for photonic devices. Both technologies have been successfully adopted in the commercial market. However, there is still room for further improvement in terms of performance. For example, the photonic systems for displays or optoelectronic communication typically include light-emitting elements, optics, and Si-based drivers. With the conventional approaches, the operating speed is limited and process complexity at die level is indispensable. In order to reduce the complexity of process and improve the switching speed, many research teams are interested in monolithic integration of GaN-based HEMTs and LEDs in recent years, which possesses advantages of higher switching speed, better reliability, and higher efficiency [1,2,3,4,5,6]. In general, there are two main approaches to integrate HEMTs and LEDs on a single chip. One is selective area growth [2,3] and the other is selective epitaxial removal [4,5,6]. Both of these methods rely on a metal interconnection between the drain region and the light-emitting region to bridge the electronic function and the optical function. However, this metal interconnection may introduce excess parasitic effects when the device is operated at high frequencies. In order to simplify the processes, and reduce the cost and the parasitic effects from the metal interconnection, an AlGaN/GaN HEMT structure is demonstrated in our previous works that includes a built-in light emitter, named a light-emitting HEMT(LE-HEMT). Different from typical LEDs, an LE-HEMT uses radiative recombination of holes from p-GaN and electrons from 2DEG to generate light [7]. In order to improve efficiency and light intensity, a single quantum well was inserted for better electron and hole confinement [8]. However, the uniformity of light-emitting is not satisfactory and needs further improvement. In this work, LE-HEMTs were fabricated on two different epitaxy structures. The difference in these epitaxy structures is the location of the single quantum well. For better understanding of the effects of structural changes, the most important optical characteristics were predicted by simulation. Current as well as electron and hole distributions, and radiative recombination rates at different biases and locations, were investigated by a software two-dimensional Poisson and drift-diffusion charge control solver (2D DDCC) developed by the Optoelectronics Device Simulation Laboratory at NTU [9]. Finally, the measured optical characteristics and simulation results are compared.

## 2. Materials and Methods

**Figure 1:**
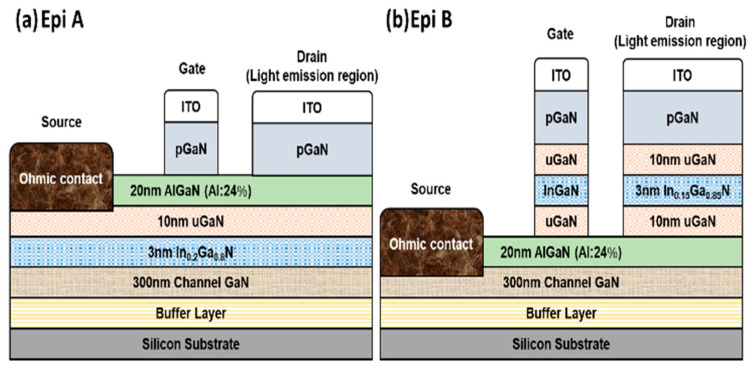
Schematic cross-sectional view of AlGaN/GaN LE-HEMTs fabricated on (**a**) Epi A and (**b**) Epi B structures.

Two different GaN-on-Si epitaxy structures used in this study were grown by metal organic chemical vapor deposition (MOCVD) with the same growth conditions. Both structures are composed of a 4.5 μm carbon doped GaN buffer layer, a 300 nm unintentionally doped GaN channel layer, a 20 nm Al_0.24_Ga_0.76_N barrier layer, a 10 nm u-GaN/3 nm InGaN/10 nm u-GaN single quantum-well layer, and an 80 nm p-GaN layer. The Mg doping concentration of the p-GaN layer is about 1 × 10^20^ cm^−3^. The difference of the two epitaxy structures is the location of the single quantum well. In Epi A, it is inserted between the AlGaN and GaN, and in Epi B it is between the p-GaN and AlGaN. Both structures were fabricated by the same processes. The fabrication processes of an LE-HEMT can be divided into five major steps. The processes started with alignment marks made by dry etch of GaN with Cl_2_/BCl_3_ mixed gas. Then, the device isolation was formed by oxygen implantation to an implant depth of around 300 nm. After that, a 225 nm ITO electrode was deposited on top of the p-GaN layer by E-beam evaporation. The ITO gate and drain electrodes were defined by lithography and the other regions were removed by wet etching. Subsequently, the p-GaN layer was selectively dry etched using photoresist as the mask to form the 2DEG region. To form Ohmic contact at the source region, Ti/Al/Ti/Au (25/125/45/55 nm) was deposited by thermal evaporator, followed by rapid thermal annealing in a nitrogen ambient at 850 °C for 30 s. A 30 nm Al_2_O_3_ was deposited as the passivation layer. Finally, windows on probing pads were opened for measurement by dry etch in SF_6_/BCl_3_ mixed gas. The schematic cross-sectional view of the two different epitaxy and device structures are shown in Figure 1.

The simulation is conducted simultaneously in this work. The parameters of structures are based on the details of previous descriptions. The electrical and optical characteristics are investigated on LE-HEMTs with L_GD_ = 5 μm and the results are described in the following section.

## 3. Results and Discussion

**Figure 2:**
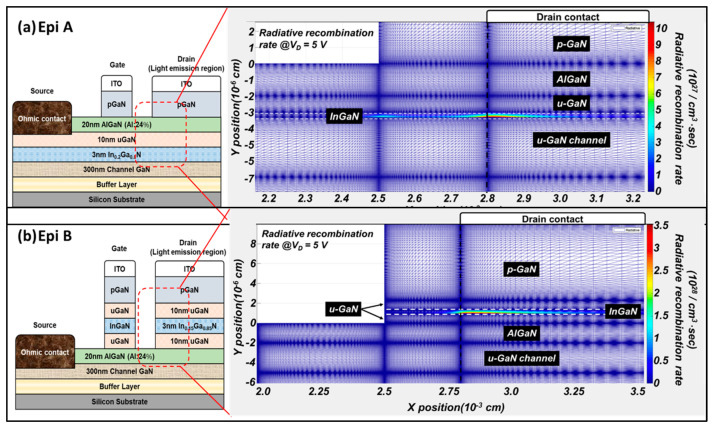
Simulation of radiative recombination rate in the light-emitting region of LE-HEMT on (**a**) Epi A and (**b**) Epi B structures.

**Figure 3:**
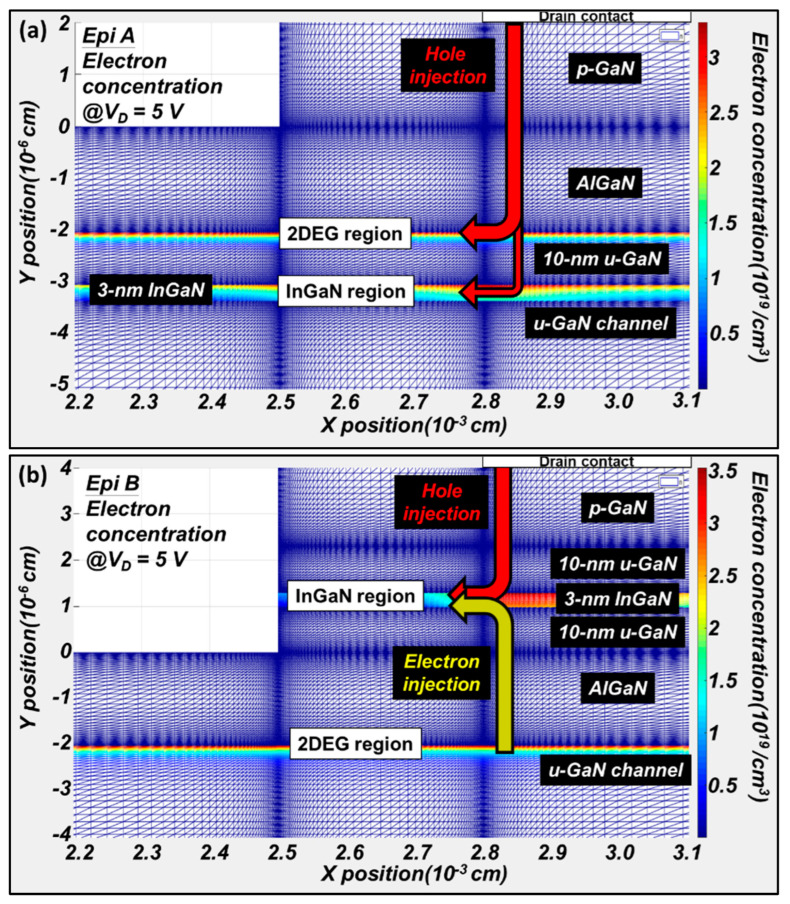
Simulation of electron concentration in the light-emitting region of LE-HEMT on (**a**) Epi A and (**b**) Epi B.

In order to explore the luminescence characteristics brought by different structures, simulations are conducted to shed some light on the important physics. The simulated radiative distribution in the light-emitting regions at I_D_ = 80 mA/mm for two devices are shown in Figure 2. In both structures, the maximum luminescence is located in the quantum well region which due to the quantum confinement of carriers. Furthermore, the details of the electron distribution are also discussed. The color in the figure represents the concentration of electrons from which two electron current paths can be found in Epi A and Epi B, as shown in Figure 3. These two current paths are located in the 2DEG region and the InGaN region separately. For both structures, the mechanism of radiative recombination is using holes from p-GaN and electrons from 2DEG/InGaN to generate light; this is indicated in the figure. In Epi A, the major hole current will first contact with the 2DEG region and emit light with 365 nm, which leads to a lower overall efficiency in the quantum well. On the contrary, the holes from p-GaN and electrons from 2DEG are better concentrated in the quantum well in Epi B at the same bias condition, yielding an improved quantum efficiency.

**Figure 4:**
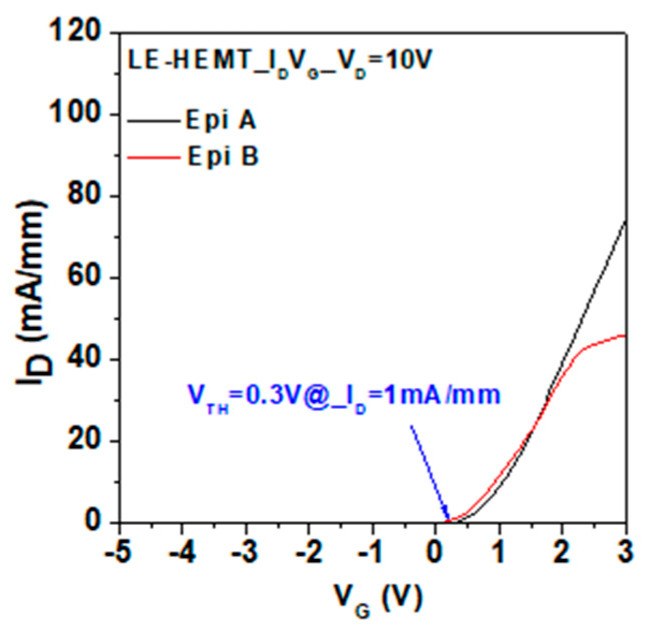
Typical I_D_-V_G_ characteristics of LE-HEMTs with both structures in this study.

**Figure 5:**
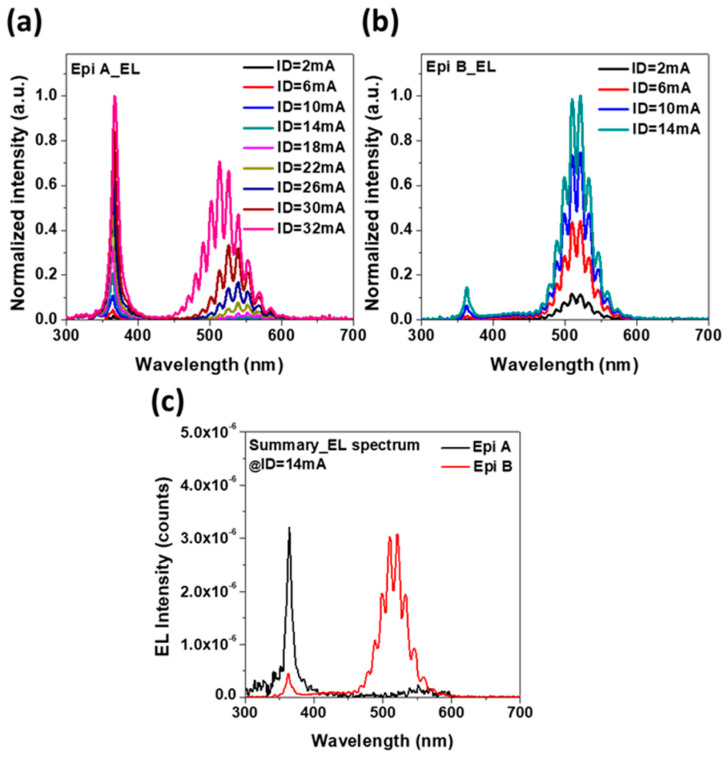
Normalized intensity of Electroluminescence spectrums of LE-HEMTs at different drain current on (**a**) epi A and (**b**) epi B. (**c**) Relative intensity of Electroluminescence spectrums of LE-HEMTs at I_D_ = 14 mA on different epitaxy structures.

Typical I_D_-V_G_ characteristics of LE-HEMTs were measured using Keysight B1505A for both structures. The result shows that the threshold voltage defined at I_D_ = 1 mA/mm is 0.3 V, as shown in Figure 4 and Figure 5, which show the electroluminescence spectrum that is measured on wafer by a spectrometer with an optical fiber on the fabricated devices. There are two main wavelength peaks that can be found in spectrums on both structures. One is the 365 nm peak which is attributed to the energy bandgap of GaN. The other is the 525 nm peak which is attributed to the recombination of electrons and holes confined in the InGaN quantum well. According to the experiment results, Epi A shows a stronger peak of 365 nm in UV region, which is owing to the current path flowing through the 2DEG channel. At high current level, a blue shift can be found in Epi A, which is attributed to the Quantum Confined Stark Effect (QCSE) [10]. Different from Epi A, Epi B shows a major peak at 525 nm, which indicates better radiative recombination in the InGaN quantum well due to much-improved electron confinement and transportation in the well. Figure 5c shows the comparison of absolute luminescence in two different structures at the same current condition. The result shows a shift of maximum wavelength which implies a better quantum confinement in the quantum well in Epi B. Both of these findings are consistent with the simulations.

**Figure 6:**
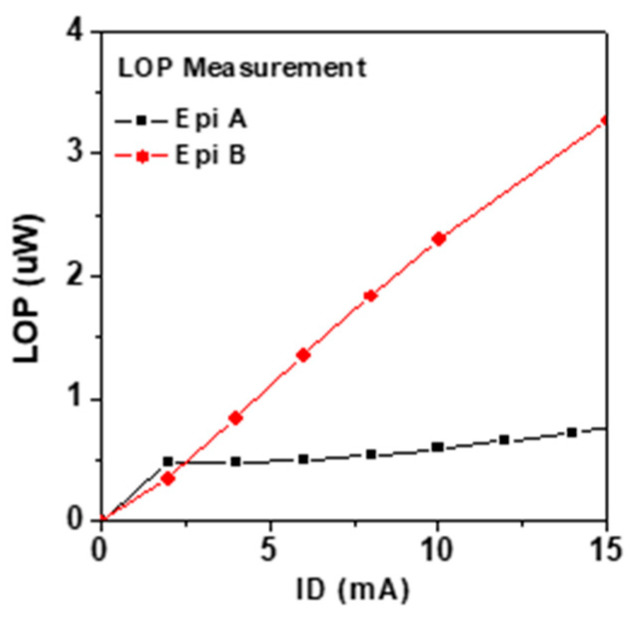
The characteristic of light output power versus I_D_ measured on LE-HEMTs on two epitaxy structures.

**Figure 7:**
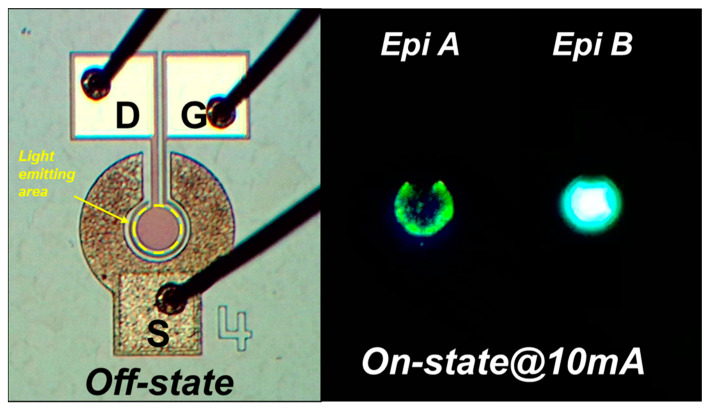
Images of a fabricated LE-HEMT under microscope and light emission when the device is switched on.

Fabricated LE-HEMTs were wire bonded and packaged in DIP-48 for optical measurements. Figure 6 shows the light output power measured with an integrating sphere on both structures. The light output power of Epi A is lower than that of Epi B due to the separation of current paths which leads to a lower efficiency in quantum well region. This is again consistent with the prediction by simulation. The images of electroluminescence of both LE-HEMTs taken by an optical microscope are depicted in Figure 7. The LE-HEMTs were biased at V_G_ = 3 V and I_D_ = 10 mA. At the same bias condition, Epi B shows great improvement in the uniformity of emitted light which is due to better confinement of electrons in the quantum well.

## 4. Conclusions

In this study, AlGaN/GaN light-emitting HEMTs with a single quantum well inserted in different locations in epitaxy layers were fabricated and analyzed. The experiment results show that the epi structure, with the quantum well inserted underneath the AlGaN barrier instead of above, has better luminescence uniformity and quantum efficiency. In addition, these optical characteristics improvements are confirmed by simulation as explained by the electron and hole current paths in the device. Further improvement in optical characteristics can be expected for the proposed LE-HEMT in the near future by refining the growth and process conditions as well as geometry and compositions of the device.
